# Retinal Nerve Fiber Layer and Macular Thickness in Parkinson's Disease Patients

**DOI:** 10.7759/cureus.16224

**Published:** 2021-07-07

**Authors:** Muhamad Ruzaini Abd Hamid, Wan-Hazabbah Wan Hitam, Sanihah Abd Halim

**Affiliations:** 1 Department of Ophthalmology, Hospital Tuanku Fauziah, Kangar, MYS; 2 Department of Ophthalmology and Visual Science, School of Medical Sciences, Universiti Sains Malaysia, Kota Bharu, MYS; 3 Department of Neurology, School of Medical Sciences, Universiti Sains Malaysia, Kota Bharu, MYS

**Keywords:** pakinson's disease, rnfl thickness, macular thickeness, neurodegenerative disease, optical coherent tomography

## Abstract

Objective: This study aimed to evaluate the retinal nerve fiber layer (RNFL) thickness and macular thickness in Parkinson’s disease (PD) patients.

Methods: The present study is a comparative cross-sectional, hospital-based study. A total number of 64 PD patients and 64 controls were recruited. Candidates that fulfilled the criteria with normal ocular examinations were undergone optical coherent tomography (OCT) examinations of the right eye. RNFL and macular thickness were evaluated.

Results: There was a statistically significant reduction in RNFL thickness in average (adjusted mean 84.32 vs 95.93, p ≤ 0.001), superior (adjusted mean 105.15 vs 118.13, p ≤ 0.010), and inferior (adjusted mean 104.95 vs 126.55, p ≤ 0.001) PD group compared to the control group. The macula thickness also was significantly reduced in average (adjusted mean 266.51 vs 281.34, p = 0.015), central (adjusted mean 236.37 vs 255.55, p = 0.001), outer superior (adjusted mean 269.16 vs 278.19, p = 0.014), outer inferior (adjusted mean 256.34 vs 272.24, p ≤ 0.001), and outer nasal (adjusted mean 287.64 vs 302.84, p = 0.001) PD group compared to the control group. There was a significant positive correlation between RNFL thickness and visual acuity among PD patients in the inferior segment with p = 0.020 and nasal segment with p ≤ 0.001. There was also a significant positive correlation between macular thickness and visual acuity among PD patients in the inner temporal segment with p = 0.006, outer superior segment with p = 0.003, and outer temporal segment with p ≤ 0.001.

Conclusion: The mean of the average, superior, and inferior RNFL thickness was significantly lower in the PD group compared to the control. The mean of the average, central, outer superior, outer inferior, and outer nasal macular thickness was significantly lower in the PD group compared to the control.

## Introduction

Parkinson’s disease (PD) is a progressive, neurodegenerative disorder of motor neurons characterized by the motor symptoms of bradykinesia, tremor, rigidity, and postural stability [1]. It was first described as “shaking palsy” by James Parkinson in 1817. However, it has been progressively linked with the involvement of other systems namely autonomic, olfactory, and visual systems [2]. PD is caused by the loss of dopaminergic neurons in the basal ganglia-substantia nigra pars compacta of the midbrain [3]. It is more common in older populations, affecting about 1% of people aged more than 60 years. PD affects one to two per 1000 of the population at any time [4]. The Malaysian Parkinson’s Disease Association estimated that about 15,000 to 20,000 patients suffer from PD in January 2012. It has been estimated that the number of PD patients in the world will double by the year 2030 [5].

Visual symptoms are common in PD, including difficulty to read, double vision, feelings of presence and passage in the visual periphery, and complex visual hallucinations [6]. Although these symptoms likely result from central visual processing deficits, lower level disturbances of visual function such as at the retinal or macular levels may also contribute to visual dysfunction in PD [7]. Dopamine dysfunction in PD is seen not only in basal ganglia but also in the retina, particularly in the horizontal, amacrine, bipolar, and ganglion cells. However, it remains unknown whether the visual dysfunctions in PD patients are related to the structural alteration of the retina.

Several studies carried out before to detect the difference in retinal nerve fiber layer (RNFL) thickness in PD patients compared to normal populations. Unfortunately, the results have not always been consistent. Some studies demonstrated a significant reduction in RNFL thickness among PD patients [8-11]. Some studies, however, reported that there is no difference in RNFL thickness between those two groups [7,12,13]. Controversial results may be predetermined by the subject selection, small sample sizes, or variable sensitivity of optical coherent tomography (OCT) instruments. Analysis of macular thickness in PD has also been studied previously, usually in conjunction with the analysis of RNFL thickness. Some studies suggested significantly diminished macular thickness in PD patients [3,14,15].

PD is a degenerative disease affecting primarily the central nervous system. The human retina is also considered a part of the central nervous system in the human body. Thus, we would expect anatomical changes of the retina in patients affected by PD. Until now, there is no single test with sufficient sensitivity and specificity to reliably diagnose PD. The diagnosis is based on the history and careful neurologic examinations. This study is to evaluate the changes in RNFL thickness and macular thickness in PD patients compared to normal populations. RNFL and macular thickness could be useful parameters to diagnose PD.

## Materials and methods

This comparative cross-sectional study was conducted between October 2017 and March 2019. This study obtained ethical approval from the Human Research Ethics Committee, Universiti Sains Malaysia (USM/JEPeM/17070323) and was conducted in accordance with the Declaration of Helsinki for human research.

Patient selection

Recruitment of PD patients was conducted in the Neuromedical Clinic, Universiti Sains Malaysia Hospital. The sample size was calculated via Power and Sample (PS) Software 2010 (Vanderbilt University School of Medicine, Nashville, TN) and G*Power 3.1.9.2 (Heinrich-Heine-Universität Düsseldorf, Germany) by using Kirbas et al. [10]. A total of 64 PD patients were recruited. Those patients were known cases of PD on treatment that met the criteria for diagnosis of PD after proper medical history review and physical examination done based on UK Brain Bank criteria. Only PD patients who able to communicate and ambulate and able to undergo examinations in the clinic such as visual acuity testing, slit-lamp examination, and OCT were selected. The control group consists of 64 individuals aged more than 50 years old who presented to the Ophthalmology Clinic. Only PD patients and controls without impaired media opacity, including corneal scar, significant cataract, and vitreous opacity, that affect the quality of OCT image were included in this study. Patients who had a pre-existing optic neuropathy, retinopathy, maculopathy, history of trauma or previous ocular surgery, and systemic disease of cerebral vascular accident, intracranial lesion, neurological and demyelinating diseases were excluded. All participants who consented to take part in the study undergo visual acuity assessment, thorough ocular examinations, and fundus evaluation via slit-lamp biomicroscopy (Topcon Corp, Japan). Intraocular pressure measurement was performed to rule out ocular pathology, which would have precluded participation in the study. All participants were then subjected to OCT examination for the right eye.

Optical coherent tomography

OCT examinations for RNFL and macular thickness were performed using the Cirrus HD SD-OCT machine (Zeiss, Germany). The tests were performed by a single and well-trained operator. Only the test or repeated test that yielded a signal strength of ≥ 6/10 was taken for interpretations to ensure the accuracy of the results. Measurements were taken on the right eye which includes average, superior, inferior, nasal, and temporal RNFL thickness, and average, central, inner superior, inner inferior, inner nasal, inner temporal, outer superior, outer inferior, outer nasal, and outer temporal of macular thickness.

Statistical analysis

Data analysis was performed using the SPSS statistical package version 22 (Chicago, IL: SPSS Inc.). Descriptive analysis was used for the mean values and SD. All values will be tested for normal distribution in both groups. For demographic data, they will be tested for comparison of age, race, and gender. The Student's t-test and Pearson chi-square test were used to analyze the demographic data. All p-values of <0.05 were considered statistically significant. An independent t-test was used to compare the means of RNFL thickness and macular thickness between the study group and the control. P-value of < 0.05 was considered significant. Pearson correlation was used to determine the correlations between RNFL thickness and visual acuity, and between macular thickness and visual acuity in PD patients. The strength of association was determined and a p-value of <0.05 was considered significant. General guidelines for assigning strength of association by Cohen were used [16].

## Results

The distribution of demographic data is shown in Table 1. There were a total of 128 participants (64 PD patients and 64 age-matched controls). Among them, 65 were males while 63 were females. The age of the participants ranged from 41 to 80 years old with a mean age of 62.0±8.56 for PD patients and 59.4±6.34 for controls. A total of 100 participants were Malay, 24 were Chinese, and four were Siamese. Thirty-five participants have underlying diabetes mellitus (DM) while 60 participants have underlying hypertension (HPT) with no ocular involvement. The mean visual acuity was 0.30±0.23 for PD patients and 0.25±0.13 for controls by using the Logarithm of the Minimum Angle of Resolution (LogMAR) scale.

**Table 1:** Demographic data of Parkinson’s disease patients and controls ^a^Independent t-test ^b^Pearson chi-square test

	Parkinson (n = 64)	Controls (n = 64)	p-Value
Mean age (mean, SD)	62.0 (8.56)	59.4 (6.34)	0.060^a^
Gender (n, %)			<0.001^b^
Male	43 (67.2)	22 (34.4)	
Female	21 (32.8)	42 (65.6)	
Race (n, %)			<0.001^b^
Malay	40 (62.5)	60 (93.8)	
Chinese	20 (31.3)	4 (6.3)	
Siamese	4 (6.3)	0 (0)	
Diabetes mellitus			0.029^b^
Yes	12 (18.8)	23 (35.9)	
No	52 (81.3)	41 (64.1)	
Hypertension			0.479^b^
Yes	28 (43.8)	32 (50.0)	
No	36 (56.3)	32 (50.0)	
Visual acuity (mean, SD)	0.30 (0.23)	0.25 (0.13)	0.003^a^

The mean RNFL thickness of PD patients and controls are shown in Table 2. We observed a decrease in the mean RNFL thickness of all quadrants in the PD group compared to the control group. There was a statistically significant reduction in average RNFL thickness with p = 0.035, inferior RNFL thickness with p ≤ 0.001, and nasal RNFL thickness with p = 0.046 in the PD group compared to the control group.

**Table 2:** Mean RNFL thickness in Parkinson’s disease patients and controls ^a^Independent t-test (p < 0.05) RNFL: retinal nerve fiber layer

RNFL	Mean (SD)		Mean Differences (95% CI)	T-statistic (df)	p-Value^a^
	Parkinson (n = 64 )	Controls (n = 64 )			
Average	84.31 (16.51)	95.94 (14.77)	-11.63 (-17.10, -6.15)	-4.199 (126)	0.035
Superior	105.75 (27.02)	117.53 (24.99)	-11.78 (-20.89, -2.68)	-2.561 (125.24)	0.906
Inferior	105.00 (37.55)	126.5 (18.95)	-21.5 (-31.91, -11.10)	-4.089 (126)	<0.001
Nasal	64.83 (12.99)	73.03 (27.20)	-8.20 (-15.66, -0.75)	-2.177 (126)	0.046
Temporal	61.77 (16.81)	66.38 (16.81)	-4.61 (-10.21, 0.99)	-1.629 (124.69)	0.584

The mean RNFL thickness between PD patients and controls after controlling potential confounders is shown in Table 3. We observed a decrease in the mean RNFL thickness of all quadrants in the PD group compared to the control group after controlling potential confounders, such as gender, DM, and HPT. There was a statistically significant reduction in average RNFL thickness (adjusted mean 84.32 vs 95.93, p ≤ 0.001), superior RNFL thickness (adjusted mean 105.15 vs 118.13, p = 0.010), and inferior RNFL thickness (adjusted mean 104.95 vs 126.55, p ≤ 0.001) in the PD group compared to the control group.

**Table 3:** Mean RNFL thickness in Parkinson’s disease patients and controls after controlling potential confounders ^a^Adjusted mean using ANCOVA after controlling for gender, DM, and HPT (p < 0.05). RNFL: retinal nerve fiber layer; ANCOVA: analysis of covariance; DM: diabetes mellitus; HPT: hypertension

RNFL	Adj Mean		Adj Mean Differences (95% CI)	F-statistic (df)	p-Value^a^
	Parkinson (95% CI)	Controls (95% CI)			
Average	84.32 (80.22, 88.42)	95.93 (91.83, 100.03)	-11.61 (-17.66, -5.56)	14.421 (1, 123)	<0.001
Superior	105.15 (98.47, 111.84)	118.13 (111.44, 124.81)	-12.97 (-22.84, -3.11)	6.780 (1, 123)	0.010
Inferior	104.95 (97.21, 112.70)	126.55 (118.81, 134.29)	-21.59 (-33.01, -10.17)	14.008 (1,123)	<0.001
Nasal	66.14 (60.68, 71.61)	71.72 (66.25, 77.18)	-5.57 (-13.64, 2.49)	1.870 (1, 123)	0.174
Temporal	61.17 (57.01, 65.33)	66.97 (62.81, 71.13)	-5.80 (-11.93, 0.34)	3.498 (1, 123)	0.064

The mean macular thickness of PD patients and controls are shown in Table 4. We observed a decrease in the mean macular thickness in the PD group compared to the control group in all quadrants except inner inferior and inner nasal quadrants. There was a significant reduction in the mean of average macular thickness with p = 0.021, inner superior macular thickness with p ≤ 0.007, inner temporal macular thickness with p = 0.002, outer superior macular thickness with p = 0.047, and outer temporal macular thickness with p = 0.045 in the PD group compared to the control group.

**Table 4:** Mean macular thickness in Parkinson’s disease patients and controls ^a^Independent t-test (p < 0.05)

Macula	Mean (SD)		Mean Differences (95% CI)	T-statistic (df)	p-Value^a^
	Parkinson (n = 64 )	Controls (n = 64 )			
Average	270.13 (43.37)	277.72 (14.74)	-7.59 (-18.93, 3.74)	-1.326 (126)	0.021
Centre	240.25 (38.65)	251.67 (22.42)	-11.42 (-22.50, -0.34)	-2.045 (101.08)	0.155
Inner superior	314.72 (31.56)	316.56 (16.28)	-1.84 (-10.63, 6.94)	-0.415 (126)	0.007
Inner inferior	312.53 (30.56)	311.98 (18.33)	-0.55 (-8.27, 9.36)	0.123 (126)	0.010
Inner nasal	318.94 (32.44)	316.92 (25.71)	2.02 (-8.23, 12.26)	0.390 (119.76)	0.158
Inner temporal	300.81 (29.92)	304.14 (17.76)	-3.33 (-11.94, 5.28)	-0.765 (126)	0.002
Outer superior	269.88 (21.12)	277.47 (15.85)	-7.59 (-14.13, -1.06)	-2.301 (126)	0.047
Outer inferior	258.50 (19.25)	270.08 (16.49)	-11.58 (-17.85, -5.31)	-3.654 (123.11)	0.204
Outer nasal	291.34 (29.63)	299.14 (19.14)	-7.80 (-16.54, 0.94)	-1.768 (107.76)	0.201
Outer temporal	259.75 (30.02)	260.73 (20.56)	-0.98 (-9.98, 8.02)	-0.216 (126)	0.045

The mean macular thickness of PD patients and controls after controlling potential confounders is shown in Table 5. We observed a decrease in the mean macular thickness of all quadrants in the PD group compared to the control group after controlling potential confounders, such as gender, DM, and HPT. There was a statistically significant reduction in average macular thickness (adjusted mean 266.51 vs 281.34, p = 0.015), central macular thickness (adjusted mean 236.37 vs 255.55, p = 0.001), outer superior macular thickness (adjusted mean 269.16 vs 278.19, p = 0.014), outer inferior macular thickness (adjusted mean 256.34 vs 272.24, p ≤ 0.001), and outer nasal macular thickness (adjusted mean 287.64 vs 302.84, p = 0.001) in the PD group compared to the control group.

**Table 5:** Mean macular thickness in Parkinson’s disease patients and controls after controlling potential confounders ^a^Adjusted mean using ANCOVA after controlling for gender, DM, and HPT (p < 0.05). ANCOVA: analysis of covariance; DM: diabetes mellitus; HPT: hypertension

Macula	Adj Mean		Adj Mean Differences (95% CI)	F-statistic (df)	p-Value^a^
	Parkinson (95% CI)	Controls (95% CI)			
Average	266.51 (258.47, 274.54)	281.34 (273.31, 289.37)	-14.83 (-26.68, -2.99)	6.143 (1, 123)	0.015
Centre	236.37 (228.53, 244.21)	255.55 (247.71, 263.40)	-19.18 (-30.76, -7.61)	10.767 (1, 123)	0.001
Inner superior	311.39 (305.17, 317.61)	319.89 (313.67, 326.11)	-8.50 (-17.68, 0.68)	3.361 (1, 123)	0.069
Inner inferior	308.85 (302.73, 314.96)	315.67 (309.56, 321.78)	-6.82 (-15.84, 2.20)	2.242 (1, 123)	0.137
Inner nasal	315.53 (308.19, 322.87)	320.23 (312.99, 327.67)	-4.80 (-15.63, 6.03)	0.769 (1, 123)	0.382
Inner temporal	299.41 (293.05, 305.77)	305.54 (299.18, 311.90)	-6.13 (-15.52, 3.25)	1.674 (1, 123)	0.198
Outer superior	269.16 (264.32, 273.99)	278.19 (273.35, 283.02)	-9.03 (-16.16, -1.90)	6.277 (1, 123)	0.014
Outer inferior	256.34 (251.89, 260.79)	272.24 (267.79, 276.69)	-15.90 (-22.47, -9.34)	22.98 (1, 123)	<0.001
Outer nasal	287.64 (281.58, 293.71)	302.84 (296.78, 308.91)	-15.20 (-24.14, -6.26)	11.315 (1, 123)	0.001
Outer temporal	257.09 (250.62, 263.55)	263.40 (256.93, 269.86)	-6.31 (-15.85, 3.23)	1.715 (1, 123)	0.193

Table 6 shows the correlation between RNFL thickness and visual acuity among PD patients. There was a significant weak positive correlation in the inferior quadrant with p = 0.020 and a significant moderate positive correlation in the nasal quadrant with p ≤ 0.001. There were also weak negative correlations in the superior and temporal quadrants but they were not statistically significant. 

**Table 6:** Correlation between RFNL thickness and visual acuity in Parkinson’s disease patients ^a^Pearson’s correlation (p < 0.05) RNFL: retinal nerve fiber layer

RNFL	Pearson’s Correlation	p-Value^a^
Average	0.191	0.131
Superior	-0.143	0.260
Inferior	0.290	0.020
Nasal	0.441	<0.001
Temporal	-0.010	0.937

Table 7 shows the correlation between macular thickness and visual acuity among PD patients. There was a significant weak positive correlation in average with p = 0.035 and significant moderate positive correlations in the inner temporal quadrant with p = 0.006, outer superior quadrant with p = 0.003, and outer temporal quadrant with p ≤ 0.001.

**Table 7:** Correlation between macular thickness and visual acuity in Parkinson’s disease patients ^a^Pearson’s correlation (p < 0.05)

Macula	Pearson’s Correlation	p-Value^a^
Average	0.264	0.035
Centre	0.073	0.565
Inner superior	0.154	0.224
Inner inferior	0.123	0.334
Inner nasal	0.213	0.092
Inner temporal	0.338	0.006
Outer superior	0.368	0.003
Outer inferior	0.150	0.238
Outer nasal	0.112	0.377
Outer temporal	0.428	<0.001

## Discussion

PD is caused by the loss of dopaminergic neurons in the basal ganglia of the midbrain [3]. Visual symptoms are common in PD that include difficulty to read, double vision, feelings of presence and passage in the visual periphery, and complex visual hallucinations [6]. Although these symptoms likely result from central visual processing deficits, they may also be contributed by the disturbance at the retina or macular levels. It is possible because the intrinsic dopaminergic neurons have been identified in the retinas of different animal species, including humans [17]. Dopamine dysfunction in PD is seen not only in basal ganglia but also in the retina, particularly in the horizontal and amacrine, bipolar, and ganglion cells. An autopsy study that was conducted on eight PD patients found reduced dopamine levels in the retina [18]. However, it remains unknown whether the visual dysfunctions in PD patients are related to the structural alteration of the retina.

The mean (SD) age of PD patients in this study is 61.97 (8.56) years. This is fairly consistent with several other studies [3,9,10]. The majority of the participants in our study are Malays. This is because our study was done in Kelantan, which has a predominantly Malay population in the north-eastern state of Peninsular, Malaysia. Thirty-five participants in our study have underlying diabetes mellitus (DM) while 60 participants have underlying hypertension (HPT). Systemic risk factors for RNFL and macular defect include DM and HPT [19-21]. These risk factors have been included in our analysis of covariance (ANCOVA) analysis as potential cofounders. The mean visual acuity was significantly lower in PD patients compared to controls (p = 0.003).

Our result showed a reduced mean RNFL thickness in all four quadrants in the PD group compared to controls. It was statistically significant in the average thickness with a mean difference of -11.61 (p ≤ 0.001), superior thickness with a mean difference of -12.97 (p = 0.010), and inferior thickness with a mean difference of -21.59 (p ≤ 0.001) after controlling potential cofounders. This is similar to the earliest study by Inzelberg et al., who first reported significant inferotemporal peripapillary RNFL losses in 10 PD patients [9]. Moschos et al. also found significant RNFL thinning in inferior and temporal areas [22]. Kirbas et al. studied a larger number of PD patients also found similar findings. They found that the RNFL thickness among idiopathic PD patients was thinner as compared to healthy controls especially in the temporal quadrant [10]. They also postulated that the RNFL thinning in PD patients may be attributed by the depletion of dopamine in the retina. However, some studies reported that there was no difference in RNFL thickness between those two groups. A study by Tsironi et al. stated that there is no difference in RNFL thickness in PD patients even when they demonstrate glaucomatous-like perimetric defects [13]. Archibald et al. reported that despite a reduction in both visual acuity and contrast sensitivity in PD patients, there is no difference in retinal thickness detected between those two groups [7]. The comparison between these studies is summarised in Table 8 and Table 9.

**Table 8:** Comparison of studies on RNFL thickness in Parkinson’s disease patients PD: Parkinson’s disease; C: controls; NR: not reported; OCT: optical coherent tomography; RNFL: retinal nerve fiber layer

Author	Year	Country	OCT Model	No. of Patients		Mean Age	
				PD	C	PD	C
Present study	2021	Malaysia	Cirrus HD	64	64	62.0	59.4
Inzelberg et al. [9]	2004	Israel	NR	10	10	64.0	67.0
Moschos et al. [22]	2011	Greece	Stratus Model 3000	16	20	57.0	52.0
Kirbas et al. [10]	2013	Turkey	Cirrus HD	42	40	59.3	57.0
Tsironi et al. [13]	2012	Greece	Stratus	24	24	66.6	64.3
Archibald et al. [7]	2011	United Kingdom	Stratus Model 3000	34	17	71.6	71.3

**Table 9:** Results of studies on mean RNFL thickness in Parkinson’s disease patients PD: Parkinson’s disease; C: controls; NR: not reported; RNFL: retinal nerve fiber layer

Author	Average			Superior			Inferior			Nasal			Temporal		
	PD	C	p-Value	PD	C	p-Value	PD	C	p-Value	PD	C	p-Value	PD	C	p-Value
Present study	84.32	95.93	<0.001	105.15	118.13	0.010	104.95	126.55	≤0.001	66.14	71.72	0.174	61.17	66.97	0.064
Inzelberg et al. [9]	125.00	143.00	NR	160.00	154.00	NR	147.00	173.00	0.002	99.00	117.00	NR	101.00	126.00	0.019
Moschos et al. [22]	95.43	102.90	0.150	119.50	123.10	0.626	124.20	137.60	≤0.001	74.00	78.20	0.227	64.00	72.35	0.004
Kirbas et al. [10]	77.00	89.00	0.001	116.00	118.00	0.763	108.00	110.00	0.569	75.00	76.00	0.856	66.00	75.00	0.001
Tsironi et al. [13]	96.42	96.34	0.982	116.58	112.05	0.312	128.58	127.05	0.771	72.38	70.35	0.650	68.58	67.10	0.714
Archibald et al. [7]	89.24	83.47	0.071	102.79	90.59	0.069	118.03	116.88	0.689	73.47	63.35	0.124	62.65	62.94	0.992

Our results also showed a reduced mean macular thickness in all quadrants in the PD group compared to controls. The results were statistically significant in the average thickness with a mean difference of -14.83 (p = 0.021), central thickness with a mean difference of -19.18 (p = 0.001), outer superior thickness with a mean difference of -9.03 (p = 0.014), outer inferior thickness with a mean difference of -15.90 (p ≤ 0.001), and outer nasal thickness with a mean difference of -15.20 (p = 0.001) after controlling potential cofounders. This is similar to a study by Hajee et al., who compared the macular thickness between 23 PD patients and healthy controls using Fourier-domain OCT [3]. They found that the inner retinal layer in the macular region was significantly thinner in PD patients than in healthy subjects. They also suggested that retinal thinning may be relevant to the early diagnosis and neuroprotective treatment of PD as most of their patients were in the early stages of the disease. Aaker et al. found significant macular thinning in inner inferior, outer superior, and outer nasal quadrants among PD patients. However, they also reported no significant difference in RNFL thickness between PD patients and controls [23]. A large study by Satue et al. in 100 PD patients using both Cirrus HD and Spectralis OCT (Heidelberg Engineering​, Heidelberg, Germany) machines stated that there is a significant thinning in RNFL as well as macular thickness in PD patients [14]. Another study by Ahn et al. also found that retinal thinning was present in the early stages of PD and this correlated with disease severity [8]. The retinal thinning may be linked to nigral dopaminergic degeneration. In addition, a recent study by Sengupta et al. stated that the macular volumes in PD patients were diminished in both perifoveal and outer macular regions with preserved foveal volume [15]. They also stated that the correlation between the dopaminergic cells and RNFL loss is still uncertain. This is because the currently available OCT software is not able to determine the thickness of the different retinal layers. However, some studies reported that there was no macular thinning in PD. Kopal et al. did not show any significant difference in RNFL thickness and macular thickness between PD patients and healthy controls [12]. They concluded that functional and structural changes in the retina are not related to visual symptoms in PD. The comparison between the studies on macular thickness in PD patients is summarised in Table 10 and Table 11.

**Table 10:** Comparison of studies on macular thickness in Parkinson’s disease patients PD: Parkinson’s disease; C: controls; NR: not reported; OCT: optical coherent tomography

Author	Year	Country	OCT Model	No. of Patients		Mean Age	
				PD	C	PD	C
Present study	2021	Malaysia	Cirrus HD	64	64	62.0	59.4
Hajee et al. [3]	2009	America	Fourier-domain	24	17	64.0	63.5
Aaker et al. [23]	2010	America	Spectralis	9	16	64.0	67.0
Satue et al. [14]	2013	Spain	Cirrus HD and Spectralis	100	100	64.0	64.0
Ahn et al. [8]	2018	South Korea	Spectralis	49	54	68.9	70.6
Sengupta et al. [15]	2018	India	Spectralis	34	50	64.9	67.3
Kopal et al. [12]	2012	Czech Republic	Stratus Model 3000	44	15	69.9	NR

**Table 11:** Results of studies on mean macular thickness in Parkinson’s disease patients

Author	Results	p-Value
Present study	Thinning in average, center, outer superior, outer inferior, and outer nasal quadrants	0.015, 0.001, 0.014, <0.001, 0.001
Hajee et al. [3]	Thinning of paramacular inner retinal layer	0.010
Aaker et al. [23]	Thinning in inner inferior, outer superior, and outer nasal quadrants	0.001, 0.026, 0.016
Satue et al. [14]	Cirrus HD = Thinning in the center and outer inferior quadrants; Spectralis = Thinning in the center, inner inferior, inner temporal, and outer inferior quadrants	0.030, 0.019, 0.034, 0.014, 0.045, 0.050
Ahn et al. [8]	Thinning in inner inferior and inner temporal quadrants	0.009
Sengupta et al. [15]	Thinning in all quadrants with preserved central volume	<0.001
Kopal et al. [12]	No significant thinning	0.710

Controversial results in RNFL and macular thickness in previous studies may be predetermined by the subject selection, such as the duration of illness, stage of the disease, and compliance to treatment. Other factors include small sample sizes and variable sensitivity of OCT instruments [3,7-15].

Our correlation analysis between RNFL thickness and visual acuity among PD patients showed a significant weak positive correlation in the inferior quadrant (p = 0.020) and a significant moderate positive correlation in the nasal quadrant (p ≤ 0.001). There were also weak negative correlations in the superior and temporal quadrants but they were not statistically significant. Our correlation analysis between macular thickness and visual acuity among PD patients showed a significant weak positive correlation in average (p = 0.035) and significant moderate positive correlations in the inner temporal quadrant (p = 0.006), outer superior quadrant (p = 0.003), and outer temporal quadrant (p ≤ 0.001). There was no negative correlation seen between macular thickness and visual acuity as compared to the correlation between RNFL thickness and visual acuity. These results can happen by chance or perhaps visual acuity reflects a better functional outcome in macular thinning compared to RNFL thinning. We could not find any literature studying the correlation between RNFL thickness and visual acuity or correlation between macular thickness and visual acuity in PD patients. However, a study by Satue et al., who reported a significant decrease in RNFL thickness and macular thickness in PD patients, found that the visual acuity was significantly reduced in PD patients compared to healthy individuals [14]. Another study by Inzelberg et al. found a reduction in visual field sensitivity among PD patients which topographically matched with the localized RNFL thinning [9]. They reported a significant reduction in inferotemporal RNFL thickness in PD patients with low-intensity visual field defect which corresponds to the RNFL thinning area. These findings strongly suggest localized RNFL defects in PD patients. 

We were unable to determine the exact duration of the PD. However, the majority of our subjects were diagnosed to have PD within six years of diagnosis when we performed an examination on them. It is possible that the greater the severity and longer duration of the PD, the greater the RNFL thickness and macular thickness alterations [8].

Limitations and recommendations

Our study was a cross-sectional study. There were no comparisons done or follow-up to actually look at the changes in our study parameters over time. We suggest that in future, a prospective study to be done on these patients in order to evaluate the possible changes in the RNFL thickness and macular thickness in these patients. We can assess whether the duration of PD can affect the RNFL and macular thickness.

Our study was limited by the presence of cognitive and motor dysfunction in our PD patients, some of whom refused to cooperate with examinations. Hoehn-Yahr staging divides PD severity into five stages. Only patients with mild to moderate PD, who were able to undergo examinations, were included in our study. We suggest that if a similar study is to be carried out in the future, the patients in the severe group should be selected and the use of more patient-friendly devices might be advocated to aid in their examinations.

Another limitation of the study is that there was no assessment of visual function except for visual acuity. We suggest that other parameters such as visual field, color vision, and contrast sensitivity be included in the study to correlate with the anatomical defect seen in RNFL and macula of PD patients.

## Conclusions

Our study showed that PD patients have significantly reduced RNFL thickness in the average, superior, and inferior quadrant compared to controls. There was also significantly reduced macular thickness in the average, center, outer superior, outer inferior, and outer nasal quadrant. There was a significant positive correlation between RNFL thickness and visual acuity among the subject in the PD group in the inferior and nasal segments. There was also a significant positive correlation between macular thickness and visual acuity among the subject in the PD group in the inner temporal, outer superior, and outer temporal segments. We believe that RNFL and macular thickness could be a useful additional parameter to diagnose PD.
